# Metabolic, Pharmacokinetic, and Activity Profile of the Liver Stage Antimalarial (RC-12)

**DOI:** 10.1021/acsomega.2c01099

**Published:** 2022-03-30

**Authors:** Yuxiang Dong, Yogesh Sonawane, Steven P. Maher, Anne-Marie Zeeman, Victor Chaumeau, Amélie Vantaux, Caitlin A. Cooper, Francis C. K. Chiu, Eileen Ryan, Jenna McLaren, Gong Chen, Sergio Wittlin, Benoît Witkowski, François Nosten, Kamaraj Sriraghavan, Dennis E. Kyle, Clemens H. M. Kocken, Susan A. Charman, Jonathan L. Vennerstrom

**Affiliations:** † College of Pharmacy, University of Nebraska Medical Center, 986125 Nebraska Medical Center, Omaha, Nebraska 68198, United States; ‡ Centre for Drug Candidate Optimisation, Monash Institute of Pharmaceutical Sciences, Monash University, 381 Royal Parade, Parkville, Victoria 3052, Australia; § Center for Tropical and Emerging Global Diseases, University of Georgia, 370 Coverdell Center, 500 D.W. Brooks Drive, Athens, Georgia 30602, United States; ∥ Department of Parasitology, Biomedical Primate Research Centre, P.O. Box 3306, 2280 GH Rijswijk, The Netherlands; ⊥ Shoklo Malaria Research Unit, Mahidol-Oxford Tropical Medicine Research Unit, Faculty of Tropical Medicine, Mahidol University, 68/30 Bantung Road, Mae Sot, Tak 63110, Thailand; # Centre for Tropical Medicine and Global Health, Nuffield Department of Medicine Research building, University of Oxford Old Road Campus, Oxford OX3 7DQ, U.K.; ∇ Malaria Molecular Epidemiology Unit, Institut Pasteur du Cambodge, 5 Boulevard Monivong, P.O. Box 983, Phnom Penh 120 210, Cambodia; ○ Department of Medical Parasitology and Infection Biology, Swiss Tropical and Public Health Institute, Socinstrasse 57, CH-4002 Basel, Switzerland

## Abstract

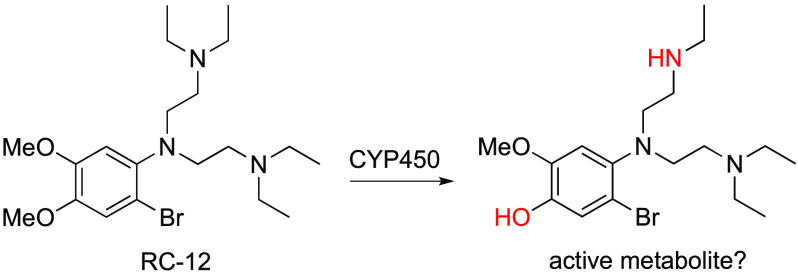

The catechol derivative RC-12 (WR 27653) (**1**) is one of the few non-8-aminoquinolines with good activity against hypnozoites in the gold-standard *Plasmodium cynomolgi*–rhesus monkey (*Macaca mulatta*) model, but in a small clinical trial, it had no efficacy against *Plasmodium vivax* hypnozoites. In an attempt to better understand the pharmacokinetic and pharmacodynamic profile of **1** and to identify potential active metabolites, we now describe the phase I metabolism, rat pharmacokinetics, and *in vitro* liver-stage activity of **1** and its metabolites. Compound **1** had a distinct metabolic profile in human vs monkey liver microsomes, and the data suggested that the *O*-desmethyl, combined *O*-desmethyl/*N*-desethyl, and *N,N*-didesethyl metabolites (or a combination thereof) could potentially account for the superior liver stage antimalarial efficacy of **1** in rhesus monkeys vs that seen in humans. Indeed, the rate of metabolism was considerably lower in human liver microsomes in comparison to rhesus monkey microsomes, as was the formation of the combined *O*-desmethyl/*N*-desethyl metabolite, which was the only metabolite tested that had any activity against liver-stage *P. vivax*; however, it was not consistently active against liver-stage *P. cynomolgi*. As **1** and all but one of its identified Phase I metabolites had no *in vitro* activity against *P. vivax* or *P. cynomolgi* liver-stage malaria parasites, we suggest that there may be additional unidentified active metabolites of **1** or that the exposure of **1** achieved in the reported unsuccessful clinical trial of this drug candidate was insufficient to kill the *P. vivax* hypnozoites.

## Introduction

**Figure 1:**
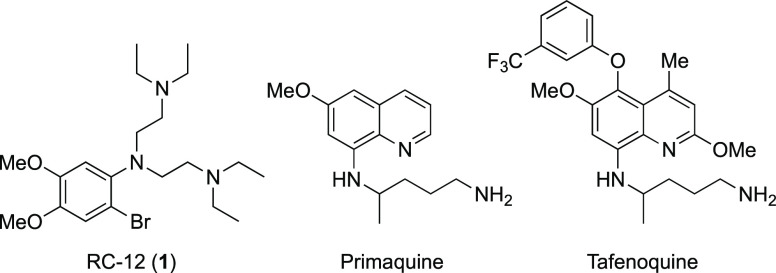
Structures of RC-12 (**1**), primaquine, and tafenoquine.

There is good momentum in the discovery and development of drugs active against the blood stage of malaria, but new compounds active against other stages of malaria are sorely needed.^1,2^ As the goal of malaria eradication is now on center stage,^3^ new drugs active against the liver stage of malaria,^4,5^ most particularly the dormant hypnozoites of *Plasmodium vivax* and *P. ovale*, will be required.^6−9^ Indeed, the prototype drug effective against hypnozoites is the 8-aminoquinoline primaquine, but this short half-life drug requires 14-day dosing to achieve radical cures, causes hemolysis and methemoglobinemia in G6PD-deficient patients, and is contraindicated in pregnancy.^6^ In 2018, tafenoquine, a next-generation 8-aminoquinoline, was approved for prophylaxis and radical cure of *P. vivax* malaria^10,11^ and appears to be a promising new drug with activity against both liver and blood stages of malaria (Figure 1). Like primaquine, tafenoquine can cause hemolytic toxicity and must be coadministered with a quantitative rapid diagnostic test for G6PD deficiency.^12^

Among the few compounds with good efficacy against hypnozoites in the gold-standard *P. cynomolgi*–rhesus monkey (*Macaca mulatta*) model, RC-12 (WR 27653) (**1**) is notable, if largely forgotten.^13^ Compound **1** (Figure 1) is about 1 order of magnitude less effective than primaquine in this model, but it is also 1 order of magnitude less toxic and produced no hemolysis.^14^ However, in a small-scale clinical trial where volunteers were infected with mosquito-borne *P. vivax* sporozoites, **1** did not prevent infections or inhibit relapses of *P. vivax*.^15^ In this trial, volunteers were given a well-tolerated 7-day course of **1** at 10 mg/(kg day), a dose regimen comparable to that which protected against and cured *P. cynomolgi* infections in the rhesus monkey model.^14^ Clearly, the dichotomy between the lack of efficacy of **1** against *P. vivax* hypnozoites in humans^15^ and the high efficacy of **1** against *P. cynomolgi* hypnozoites in rhesus monkeys^14^ points to the compelling need to better understand both the pharmacokinetic and the pharmacodynamic profile of **1** and to identify potential active metabolites that might explain these apparent species differences. We now describe our work to elucidate the Phase I metabolism using liver microsomes from various species, the pharmacokinetics in rats, and the *in vitro* liver-stage activity of **1** and its major metabolites.

## Results and Discussion

### Metabolism in Hepatic Microsomes

There was no measurable degradation of **1** (<10%), and only minor concentrations of the primary *O*-desmethyl (**2**) and *N*-desethyl (**3**) metabolites were detected when **1** (1 μM) was incubated with NADPH-supplemented human liver microsomes (0.4 mg/mL) over 1 h (Table 1). Under the same conditions, there were approximately 42% and 90% depletions of **1** upon incubation with rhesus monkey and rat liver microsomes, respectively. Two primary (**2** and **3**) and two secondary (**4** and **5**) metabolites were detected in monkey liver microsomes, whereas in rat, only one primary (**3**) and the same two secondary metabolites were detected. Subsequent studies (see below) suggested that **2** was highly unstable in rat microsomes, which may explain why it was not detected in the rat microsome incubations with **1**. The *O*-desmethyl metabolite **6** was only detected at trace concentrations in all three species, although subsequent studies (see below) indicated that this metabolite was also highly unstable in all species. The *N*-desethyl metabolite **3** appeared to be the most prominent metabolite in all species, although concentrations were very low in human microsomes. The *N,N*-didesethyl metabolite **5** was a minor metabolite in monkey microsomes and a significant metabolite in rat microsomes, and only trace concentrations were detected in human microsomes, whereas the combined *O*-desmethyl/*N*-desethyl metabolite **4** was a minor product in both rat and monkey microsomes and was not detected in human microsomes. The five metabolites for which authentic standards were available accounted for >90% of the loss of **1** in both monkey and rat liver microsomes.

**Table 1:** Metabolism of **1** upon Incubation with Hepatic Microsomes at a Substrate Concentration of 1 μM and Microsomal Protein Concentration of 0.4 mg/mL^a^

param	human	rhesus monkey	rat
CL_int_ (μL/(min mg))	<3^b^	23 ± 4.7	100 ± 2.6
average substrate depletion over a 60 min incubation (%)^c^	<10	42	90
Average Individual Metabolites Formed over a 60 min Incubation (%)^c^			
M-14 (I) *O-*demethylation (**2**)	0.2	15	ND
M-14 (II) *O-*demethylation (**6**)	ND^d^	ND	ND
M-28 *N*-deethylation (**3**)	1.6	20	64
M-42 *O-*demethylation + *N*-deethylation (**4**)	ND	2.4	6.4
M-56 (II) *N,N*-dideethylation (**5**)	ND	1.4	25
average total metabolites (%)^c^	1.8	39	95

a Incubations were conducted in the presence of NADPH at 37 °C over a 60 min period. Data represent the average of three replicate incubations. Metabolites are those for which authentic standards were available.

b <10% degradation detected; the degradation slope was not statistically different from zero (α = 0.05).

c Substrate depletion and individual and total metabolite formation data are expressed as a percentage relative to the initial concentration of the substrate.

d ND: not detected.

Additional incubations under high substrate (10 μM) and higher microsomal protein (1 mg/mL) concentrations were conducted to see if any additional metabolites could be detected. In all species, low concentrations of an M+16 metabolite were detected which appeared to represent *N*-oxidation of one of the distal tertiary amines, although the signal was too weak for a structural confirmation. An additional *N,N*-didesethyl metabolite (M-56 (I)) was also detected in monkey and rat microsomes but was only detected in trace concentrations in human microsomes.

Incubations of each of the available metabolites with microsomes from each species were also conducted using the same conditions (1 μM of the substrate, 0.4 mg/mL of the protein) (Table 2). As was seen for the parent compound, each of the metabolites with the exception of the *O*-desmethyl **6** and the *N,N*-didesethyl **5** was much more stable in human microsomes in comparison to monkey and rat microsomes. The *O*-desmethyl metabolite **2** was highly unstable in rat microsomes and, as highlighted above, possibly explains why it was not seen in incubations conducted with **1**. Similarly, the regioisomeric *O*-desmethyl metabolite **6** was very unstable in all species, and as a result, the contribution of this pathway could be underestimated.

**Table 2:** Metabolism of Authentic Metabolites of **1** upon Incubation with Hepatic Microsomes at a Substrate Concentration of 1 μM and Microsomal Protein Concentration of 0.4 mg/mL^a^

	CL_int_ (μL/(min mg))		
metabolite incubated	human	rhesus monkey	rat
M-14 (I) *O-*demethylation (**2**)	<3	8.0	264
M-14 (II) *O-*demethylation (**6**)	highly unstable in all species		
M-28 *N*-deethylation (**3**)	<3	19	24
M-42 *O-*demethylation + *N*-deethylation (**4**)	<3	12	45
M-56 (II) *N,N*-dideethylation (**5**)	6.0	7.3	4.4

a Incubations were conducted in the presence of NADPH at 37 °C over a 60 min period. Data represent a single incubation only.

**Figure 2:**
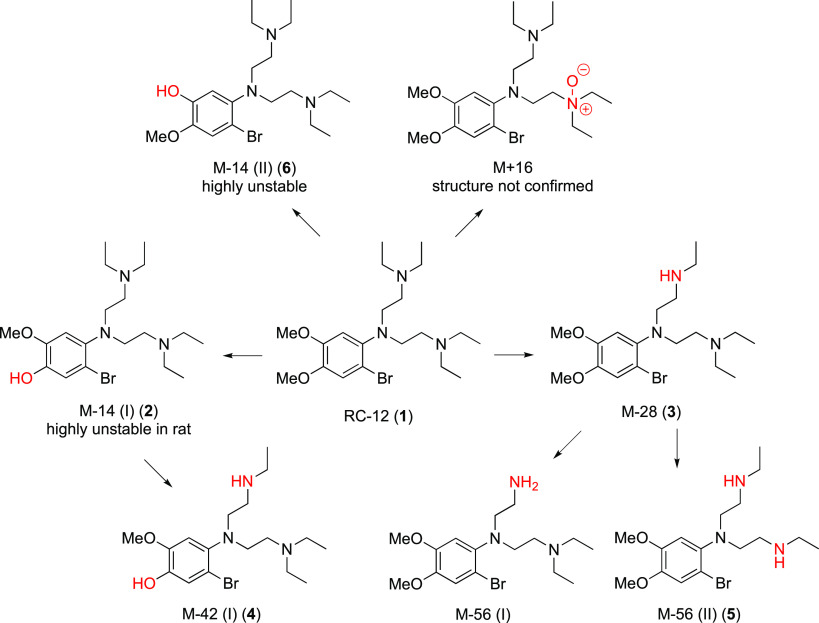
CYP450 metabolism of **1** based on data in human, monkey, and rat liver microsomes.

Overall, the metabolism studies suggested that the rate of degradation for **1** follows the order rat > monkey ≫ human and that *N*-deethylation to form **3** and subsequent metabolites is the predominant pathway. Given that metabolite **6** was highly unstable in all species and that **2** was very unstable in rat microsomes, the contributions of these pathways could be underestimated by the available data. On the basis of these results, the metabolic scheme shown in Figure 2 is proposed.

### Pharmacokinetics of **1** in Rats

**Figure 3:**
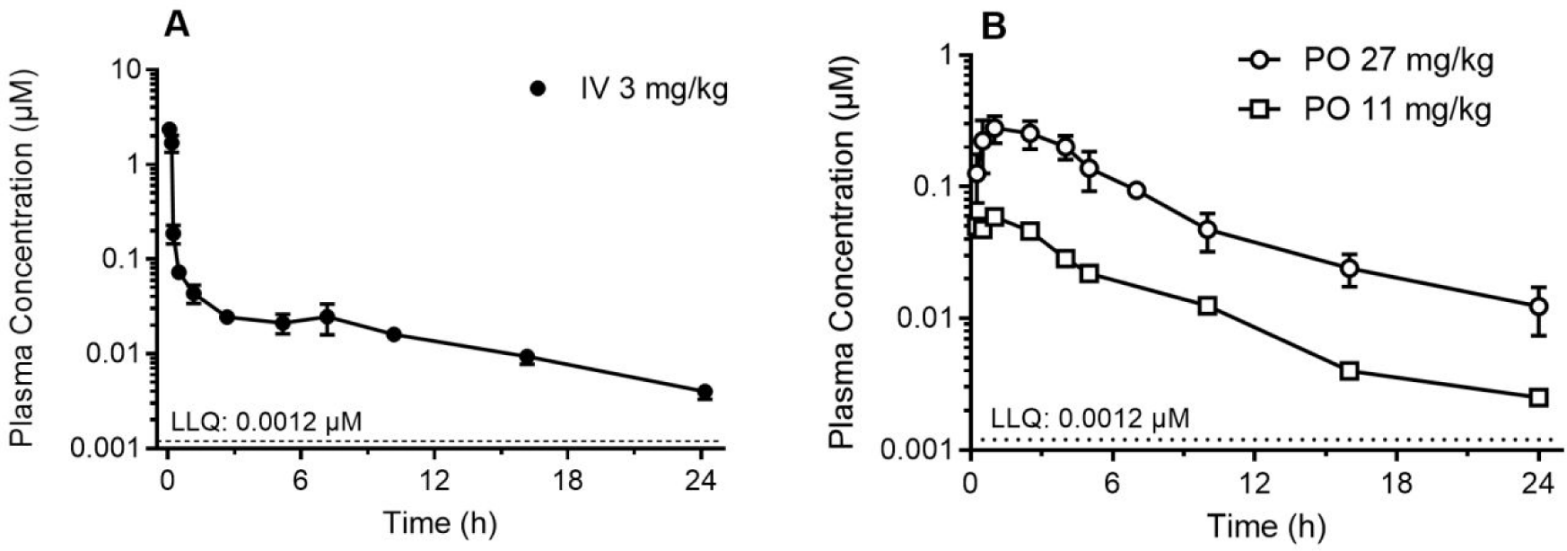
Plasma concentration versus time data for **1** in male Sprague–Dawley rats following (A) IV and (B) oral administration. Data represent the mean ± SD (*n* = 5 for 3.0 mg/kg IV, *n* = 3 for 27 mg/kg PO) and the mean (*n* = 2 for 11 mg/kg PO).

Measurable plasma concentrations of **1** were observed for the duration of the 24 h sampling period after both intravenous (IV) and oral (PO) administration (Figure 3). Compound **1** exhibited a long terminal half-life (5–7 h), a high blood to plasma ratio (2.1), a very high plasma volume of distribution (51 L/kg), and a high plasma clearance (147 mL/(min kg)) (Table 3). The high *in vivo* clearance is consistent with the high *in vitro* intrinsic clearance seen in rat microsomes and suggests that the long terminal half-life can be attributed almost entirely to the very high volume of distribution. Renal elimination accounted for approximately 10% of the overall *in vivo* clearance of **1**. Following oral administration, **1** was rapidly absorbed with maximum plasma concentrations being observed at approximately 1 h postdose and the apparent oral bioavailability was approximately 16% at 11 mg/kg and 27% at 27 mg/kg, indicating at least partial saturation of first-pass clearance pathways at the higher dose.

**Table 3:** Pharmacokinetic Parameters for **1** following IV and PO Administration to Male Sprague–Dawley Rats

	route		
	IV	PO	PO
dose (mg/kg)	3	11	27
plasma half-life (h)	7.0 ± 1.3	4.9, 6.1	7.4 ± 1.3
plasma clearance (mL/(min kg))	147 ± 33	NA^a^	NA
plasma volume of distribution (L/kg)	51 ± 10	NA	NA
dose excreted in urine as parent (%)	9.2, 10.7	NA	NA
blood:plasma	2.1	NA	NA
*C*_max_ (μM)	NA	0.07, 0.05	0.31 ± 0.03
*T*_max_ (h)	NA	1.0, 0.8	1.0
bioavailability (%)	NA	17, 15	27 ± 4

a NA: not applicable or not available.

Following IV administration of **1**, the only metabolites that were quantifiable in plasma were the deethylated metabolites **3** and **5**. Concentrations of these metabolites were present during the infusion period, indicating that they were rapidly formed, but concentrations also declined rapidly and the profiles were not well-defined. Four metabolites for which authentic standards were available (**2**–**5**) were detected in urine with the largest percentages of the **1** dose being **3** (1.7%) and **5** (1.2%). After oral administration at 27 mg/kg, there was substantial plasma exposure of both **3** and **5** with the concentrations being similar to that of the parent compound. The recovery of **3** and **5** in urine (3.3 and 3.0%, respectively, of the **1** dose) was higher than that after IV dosing, suggesting the potential for a presystemic first-pass metabolism of **1**. With the exception of an M-98 (*O*-desmethyl + *N,N*,*N*-tridesethyl) metabolite, all of the primary and secondary phase I metabolites of **1** observed in rat liver microsomes were also detected *in vivo* in rat urine. There were also a number of phase II conjugated metabolites detected, but these appeared to be secondary and tertiary conjugates of various *O*-demethylated and *N-*deethylated products.

### *In Vitro* Activity against Liver-Stage Malaria Parasites

Compound **1** and its metabolites were tested in both prophylactic and radical cure modes for activity against *P. vivax* liver-stage parasites.^16,17^ In the prophylactic assay, compounds are added the day after sporozoite invasion into hepatocytes in order to characterize the compound activity against established liver forms with a parasitophorous vacuole membrane (as opposed to activity on preinvasion sporozoites) and prior to complete maturation of hypnozoites into drug-insensitive dormant forms, which appear at about 5 days postinvasion.^18^ In the radical cure assay, compounds are added at day 5 postinfection to characterize activity against late-stage schizonts and mature PI4K inhibitor-insensitive hypnozoites.^19^ In these experiments, the threshold for activity is 75% inhibition. While **4** exhibited an IC_50_ value of 2.63 μM (*n* = 3) against schizonts in the prophylactic mode and 15.5 μM against schizonts in the radical cure mode (*n* = 2), in two independent runs, 20 μM concentrations of **1** and its other metabolites had no activity against schizonts or hypnozoites in either mode. In contrast, the control drug KDU691, a PI4K inhibitor, had IC_50_ values of 11.4 nM (schizonts) and 53.5 nM (hypnozoites) in the prophylactic mode while the control drug monensin, an ionophore, had IC_50_ values of 12.4 nM (schizonts) and 102 nM (hypnozoites) in the radical cure mode. Similarly, as previously described,^20^ 10 μM concentrations of **1** and its metabolites were also tested for activity against *P. cynomolgi* schizont (large forms) and hypnozoite (small forms) liver-stage parasites cultured in rhesus monkey hepatocytes. In these experiments, the threshold for activity is 50% inhibition. In the first run, none of the compounds had activity, except for **4**, which inhibited the growth of the schizont (large forms) by 70%. However, in a second run, all compounds were inactive. For comparison, primaquine had IC_50_ values of 1.1 and 2.7 μM against the schizont (large forms) and hypnozoite (small forms), respectively. Finally, we found that **1** at a concentration up to 15 μM had no blood stage activity against cultured *P. falciparum* (NF54 clone).

### Metabolite Synthesis

**Scheme 1:**
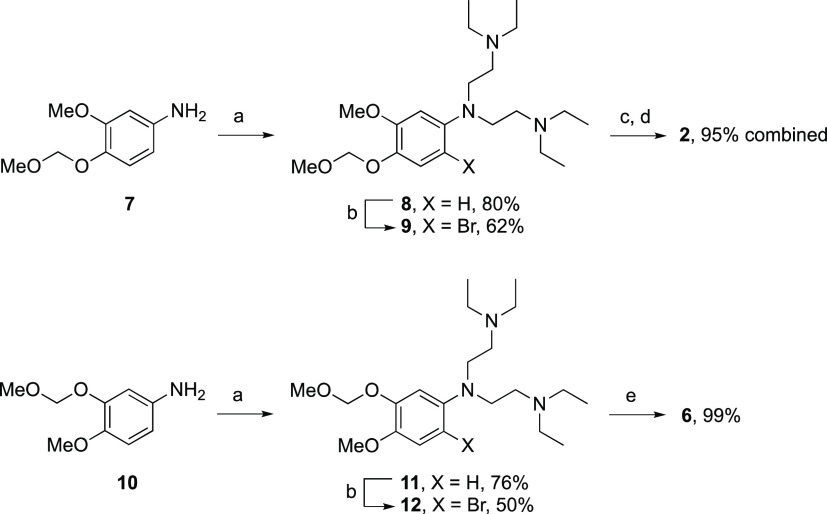
Reagents and conditions: (a) 2-diethylaminoethyl chloride HCl, K_2_CO_3_, DMF, 145 °C, 40 h; (b) Br_2_, aqueous AcOH, 0–25 °C, 14 h; (c) acetyl chloride, MeOH, −60 to +25 °C, 14 h; (d) naphthalene-1,5-disulfonic acid, Et_2_O/acetone 5/1, rt, 2 h; (e) acetyl bromide, MeOH, −60 to +25 °C, 14 h.

We obtained **1** as the bis-dihydrogen fumarate salt by following a modified procedure of Westphal.^21^ For the synthesis of the two regioisomeric *O*-desmethyl metabolites **2** and **6**, we began by running model reactions between bromo and iodo catechol ethers and secondary amines under a variety of copper- and palladium-catalyzed cross-coupling conditions,^22^ but these reactions were unsuccessful and we observed only complex mixtures of reaction products. We next investigated a possible one-step conversion of **1** to **2** and **6** via selective *O*-demethylation reactions,^23,24^ but these were likewise unsuccessful and gave incomplete conversion of **1** to multiple products. However, as depicted below, we successfully obtained **2** and **6** by bis-alkylation of the corresponding MOM-protected aniline intermediates **7**(25) and **10**(26) (Scheme 1) followed by bromination; the reversed reaction sequence failed due to steric hindrance.

**Scheme 2:**
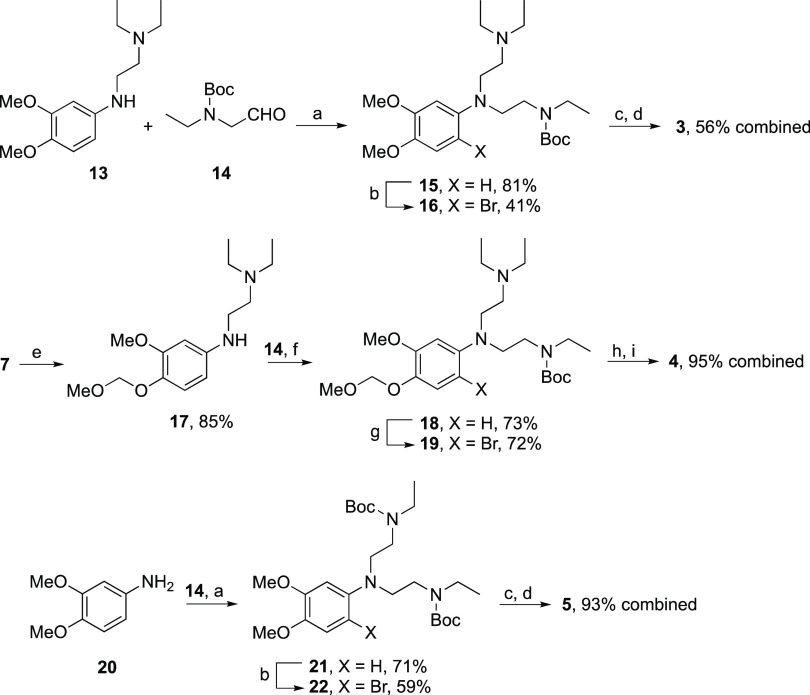
Reagents and conditions: (a) NaBH(OAc)_3_, CH_3_CN, rt, 48 h; (b) NBS, Na_2_CO_3_, DCM/H_2_O 1/1, rt, 17 h; (c) CH_3_SO_3_H, ether, rt, 24 h; (d) naphthalene-1,5-disulfonic acid, THF, rt, 1 h; (e) 2-(diethylamino)ethyl bromide hydrobromide, K_2_CO_3_, CH_3_CN, reflux, 14 h; (f) NaBH(OAc)_3_, CH_3_CN, rt, 16 h; (g) NBS, 10% aqueous acetic acid, −15 to +25 °C, 15 h, then 2 M aqueous NaOH; (h) acetyl chloride, MeOH, −60 to +25 °C, 13 h then 2 M aqueousI NaOH; (i) naphthalene-1,5-disulfonic acid, acetone, rt, 2 h.

Interestingly, if we used acetyl chloride/MeOH to generate HCl in the MOM deprotection of **12** to afford **6**, we observed (in addition to **6**) the debrominated product (ArH) and the corresponding chloride (ArCl). This outcome can be explained by the reverse and forward electrophilic aromatic substitution reactions as depicted in the following equations:
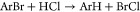

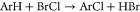
We were pleased to find that acetyl bromide/MeOH to generate HBr in the MOM deprotection worked well to afford **6** as the trihydrobromide salt in high yield. As illustrated by the syntheses of metabolites **3**–**5**, we used a reductive amination approach with the Boc-protected aldehyde **14** with some success (Scheme 2). Metabolites **2**–**5** were isolated as their naphthalene disulfonate salts. As previously noted by Westphal^21^ and Schmidt et al.,^14^ this salt proved to be particularly useful for this compound class.

## Conclusion

To summarize, we performed the first metabolic and pharmacokinetic characterization of **1** and tested **1** and its known metabolites for *in vitro* activity against liver-stage *P. vivax* and *P. cynomolgi* malaria parasites. We hypothesized that these data might confirm that species-specific metabolism accounts for the dichotomy between the high efficacy of **1** against *P. cynomolgi* hypnozoites in rhesus monkeys and the lack of efficacy of **1** against *P. vivax* hypnozoites in humans. Compound **1** did have a distinct metabolic profile in human vs monkey (and rat) liver microsomes with considerably different rates of metabolism and extents of formation of six different phase I metabolites. Notably, the rate and extent of metabolism to the *O*-desmethyl/*N*-desethyl metabolite **4** was considerably lower in human liver microsomes in comparison to that in monkey microsomes. This was also the only metabolite to have any activity against liver-stage *P. vivax*; however, it was not consistently active against liver-stage *P. cynomolgi*. Since **1** and the other identified phase I metabolites have no liver-stage antimalarial activity against either species and given the complexity of the metabolic profile, it is conceivable that there may be additional active metabolites of **1** that have not yet been identified. Equally, since plasma concentrations of **1** in the clinical study were not reported,^15^ it is possible that the exposure of **1** was insufficient to kill the *P. vivax* hypnozoites. Finally, it is conceivable that **1** and its metabolites exert liver-stage antimalarial activity via host-mediated effects. Given the similarities between the core catechol structure of **1** and the proposed 5-hydroxyprimaquine active metabolite^6,27^ of primaquine, these data could also inform ongoing investigations to understand and separate efficacy and hemolytic toxicity in the 8-aminoquinoline drug class.

## Methods

### Synthesis

Melting points are uncorrected. ^1^H and ^13^C NMR spectra were recorded on a 500 MHz spectrometer. All chemical shifts are reported in parts per million (ppm) and are relative to internal (CH_3_)_4_Si (0 ppm) for ^1^H and CDCl_3_ (77.0 ppm) or DMSO-*d*_6_ (39.7 ppm) for ^13^C NMR. Combustion analyses confirmed that all target compounds possessed purities of ≥95%. As indicated below, starting materials were commercially available or were prepared according to known procedures.

### *N*,*N*-Bis[2-(diethylamino)ethyl]-2-bromo-4-hydroxy-5-methoxyaniline 1,5-Naphthalenedisulfonate (**2**)

#### Step 1

To a stirred mixture of 3-methoxy-4-(methoxymethoxy)aniline (**7**;^25^ 1.16 g, 6.34 mmol) and K_2_CO_3_ (8.76 g, 63.34 mmol) in anhydrous DMF (30 mL) was added 2-diethylaminoethyl chloride hydrochloride (8.73 g, 50.7 mmol). The reaction mixture was stirred for 40 h at 145 °C. The reaction mixture was cooled to rt, poured into water (100 mL), and extracted with EA (3 × 30 mL). The organic layer was washed with water (3 × 50 mL) and dried over MgSO_4_. After removal of the solvent **in vacuo**, the residue was purified by chromatography over sg using hexane/EA/triethylamine (50/45/5) as an eluent to give *N*,*N*-bis[2-(diethylamino)ethyl]-3-methoxy-4 (methoxymethoxy)aniline (**8**; 1.93 g, 80%). ^1^H NMR (CDCl_3_): δ 6.99 (d, *J* = 9.26 Hz, 1H), 6.31 (d, *J* = 2.92, 1H), 6.18 (dd, *J* = 9.26, 2.92 Hz, 1H), 5.08 (s, 2H), 3.83 (s, 3H), 3.51 (s, 3H), 3.38 (t, *J* = 7.8 Hz, 4H), 2.54–2.60 (m, 12H), 1.04 (t, *J* = 7.3 Hz, 12H). ^13^C NMR (CDCl_3_): δ 151.1, 144.7, 137.1, 119.3, 103.4, 97.33, 96.6, 55.9, 55.6, 50.6, 50.2, 47.6, 11.9.

#### Step 2

Bromine (0.22 g, 1.35 mmol) in acetic acid (1 mL) was added dropwise with stirring over 30 min to a solution of **8** (0.4 g, 1.04 mmol) in 10% aqueous acetic acid (20 mL) at 0 °C. The mixture was stirred for 1 h at 0 °C and then for 12 h at rt. The mixture was treated with 2 M aqueous NaOH (10 mL) and then extracted with DCM (2 × 20 mL). The combined organic layers were dried over MgSO_4_, filtered, and concentrated **in vacuo**. The residue was purified by sg chromatography with hexane/EA/triethylamine (50/45/5) as eluent to afford *N*,*N*-bis[2-(diethylamino)ethyl]-2-bromo-5-methoxy-4-(methoxymethoxy)aniline (**9**; 0.30 g, 62%). ^1^H NMR (CDCl_3_): δ 7.29 (s, 1H), 6.76 (s, 1H), 5.13 (s, 2H), 3.80 (s, 3H), 3.48 (s, 3H), 3.07–3.11 (m, 4H), 2.44–2.50 (m, 12H), 0.95 (t, *J* = 7.2 Hz, 12H). ^13^C NMR (CDCl_3_): δ 149.2, 143.9, 143.2, 121.1, 112.2, 108.1, 95.8, 56.2, 55.9, 52.1, 50.8, 47.4, 11.8.

#### Step 3

To a stirred solution of **9** (0.6 g, 1.3 mmol) in MeOH (20 mL) at −60 °C was added acetyl chloride (0.41 g, 5.2 mmol). The mixture was stirred at −60 °C for 30 min and then for 12 h at rt. The mixture was concentrated, diluted with 3 M NaOH solution (10 mL), and extracted with EA (2 × 30 mL). The combined organic layers were washed with brine (50 mL), dried over MgSO_4_, filtered, and concentrated *in vacuo* to give the free base of **2** (0.52 g, 95%). ^1^H NMR (D_2_O): δ 7.06 (s, 1H), 6.72 (s, 1H), 3.78 (s, 3H), 3.07–3.10 (m, 4H), 2.49–2.53 (m, 12H), 0.98 (t, *J* = 7.3 Hz, 12H). To a solution of the free base of **2** (0.55 g, 1.31 mmol) in diethyl ether (50 mL) was added dropwise a solution of naphthalene-1,5-disulfonic acid (0.42 g, 1.44 mmol) in acetone (10 mL). The reaction mixture was stirred at rt for 2 h. The precipitate was collected by filtration, washed with acetone (2 × 25 mL), and dried at 60 °C overnight to afford **2** (0.88 g, 95%). Mp: 198–200 °C ^1^H NMR (D_2_O): δ 8.83 (d, *J* = 8.77 Hz, 2H), 8.18 (d, *J* = 7.31 Hz, 2H), 7.69 (t, *J* = 7.5 Hz, 2H), 7.18 (s, 1H), 7.02 (s, 1H), 3.83 (s, 3H), 3.28–3.34 (m, 4H), 3.06–3.10 (m, 12H), 1.12 (t, *J* = 7.3 Hz, 12H). ^13^C NMR (DMSO-*d*_6_): δ 148.02, 145.59, 143.82, 137.59, 129.68, 129.25, 124.29, 124.20, 119.20, 111.97, 110.30, 53.30, 48.42, 48.21, 47.14, 8.76. Anal. Calcd for C_29_H_42_BrN_3_O_8_S_2_: C, 49.43; H, 6.01; N, 5.96. Found: C, 49.33; H, 6.06; N, 5.87.

### *N*-[2-(Ethylamino)ethyl]-*N*-[2-(diethylamino)ethyl]-2-bromo-4,5-dimethoxyaniline 1,5-Naphthalenedisulfonate (**3**)

#### Step 1

A mixture of *N*-[2-(diethylamino)ethyl]-3,4-dimethoxyaniline (**13**; 808 mg, 3.2 mmol) and *tert*-butyl ethyl(2-oxoethyl)carbamate (**14**;1.2 g, 6.4 mmol) in CH_3_CN (20 mL) was stirred at rt for 30 min before NaBH(OAc)_3_ (1.7 g, 8.0 mmol) was added. The mixture was stirred at rt for 48 h and then concentrated *in vacuo*. The residue was diluted with saturated NaHCO_3_ (20 mL), stirred at rt for 30 min, and extracted with DCM (3 × 30 mL). The combined organic layers were washed with water (100 mL) and brine (50 mL), dried over MgSO_4_, filtered, and concentrated *in vacuo*. The crude product was purified by sg chromatography (hexanes/EA/triethylamine 60/40/5) to afford *N*-[2-[(*tert*-butoxycarbonyl)ethylamino]ethyl]-*N*-[2-(diethylamino)ethyl]-3,4-dimethoxyaniline (**15**; 1.11 g, 81%). ^1^H NMR (DMSO-*d*_6_): δ 0.95 (t, *J* = 7 Hz, 6H), 1.02 (br s, 3H), 1.38 (s, 9H), 2.47–2.51 (m, 6H), 3.11–3.29 (m, 8H), 3.63 (s, 3H), 3.73 (s, 3H), 6.18 (d, *J* = 8 Hz, 1H), 6.29 (s, 0.5H), 6.45 (s, 0.5H), 6.77 (d, *J* = 8 Hz, 1H).

#### Step 2

To a solution of **15** (1.11 g, 2.6 mmol) and Na_2_CO_3_ (1.10 g, 10.4 mmol) in DCM (40 mL) and water (40 mL) at rt was added *N*-bromosuccinimide (NBS; 600 mg, 3.37 mmol). The mixture was stirred at rt for 17 h. After separation of the organic layer, the aqueous layer was extracted with DCM (2 × 20 mL). The combined organic layers were washed with water (100 mL) and brine (50 mL), dried over MgSO_4_, filtered, and concentrated *in vacuo*. The crude product was purified by sg chromatography using hexanes/EA/TEA (60/40/4) as an eluent to afford *N*-[2-[(*tert*-butoxycarbonyl)ethylamino]ethyl]-*N*-[2-(diethylamino)ethyl]-2-bromo-4,5-dimethoxyaniline (**16**; 0.53 g, 41%). ^1^H NMR (DMSO-*d*_6_): δ 0.89 (t, *J* = 7 Hz, 6H), 0.98 (br s, 3H), 1.31–1.39 (m, 9H), 2.37–2.44 (m, 6H), 3.05–3.18 (m, 8H), 3.73 (s, 3H), 3.75 (s, 3H), 6.95 (s, 1H), 7.09 (s, 1H).

#### Step 3

To **16** (0.53 g, 1.05 mmol) was added a solution of methanesulfonic acid (1.44 g, 15 mmol) in ether (10 mL). The mixture was stirred at rt for 24 h and diluted with ether (50 mL). The ether layer was decanted to leave an oily residue at the bottom of the flask. The residue was treated with ether (50 mL), and the ether layer was decanted again. To the residue was added a solution of 1,5-naphthalenedisulfonic acid (303 mg, 1.05 mmol) in EtOH (10 mL). The mixture was stirred at rt for 1 h and concentrated *in vacuo*. To a solution of the residue in EtOH (2 mL) was added THF (10 mL). The resulting precipitate was collected by filtration and dried to afford **3** (404 mg, 56%) as a white solid. Mp: 177–179 °C. ^1^H NMR (DMSO-*d*_6_): δ 1.09 (t, *J* = 7 Hz, 6H), 1.13 (t, *J* = 7 Hz, 3H), 2.89–2.95 (m, 4H), 2.99–3.03 (m, 2H), 3.07–3.10 (m, 4H), 3.21 (t, *J* = 7 Hz, 2H), 3.31 (t, *J* = 7 Hz, 2H), 3.76 (s, 3H), 3.77 (s, 3H), 7.03 (s, 1H), 7.17 (s, 1H), 7.45 (t, *J* = 8 Hz, 2H), 7.97 (d, *J* = 7 Hz, 2H), 8.22 (s, 2H), 8.89 (d, *J* = 8 Hz, 2H), 8.90 (s, 1H). ^13^C NMR (DMSO-*d*_6_): δ 8.78, 11.22, 42.43, 43.58, 47.17, 48.03, 48.45, 50.33, 56.19, 56.21, 110.14, 112.11, 115.97, 124.25, 124.33, 129.28, 129.69, 139.27, 143.79, 147.47, 149.06. Anal. Calcd for C_28_H_40_BrN_3_O_8_S_2_·H_2_O: C, 47.45; H, 5.97; N, 5.93. Found: C, 47.69; H, 5.61; N, 5.45.

### *N*-[2-(Ethylamino)ethyl]-*N*-[2-(diethylamino)ethyl]-2-bromo-4-hydroxy-5-methoxyaniline 1,5-Naphthalenedisulfonate (**4**)

#### Step 1

To a stirred mixture of **7** (4.0 g, 21.83 mmol) and K_2_CO_3_ (9.1 g, 65.49 mmol) in acetonitrile (50 mL) was added in portions 2-(diethylamino)ethyl bromide hydrobromide (11.4 g, 43.66 mmol). After addition, the reaction mixture was heated to reflux for 14 h. The reaction mixture was cooled to rt, poured into the water (100 mL), and extracted with EA (3 × 30 mL). The combined organic layers were washed with water (3 × 50 mL) and dried over MgSO_4_. After removal of the solvent *in vacuo*, the residue was purified by sg chromatography using hexane/EA/triethylamine (50/45/5) as an eluent to give *N*-[2-(diethylamino)ethyl]-3-methoxy-4-(methoxymethoxy)aniline (**17**) as an orange oil (5.23 g, 85%). ^1^H NMR (CDCl_3_): δ 6.97 (d, *J* = 8.5 Hz, 1H), 6.25 (d, *J* = 2.5 Hz, 1H), 6.14 (dd, *J* = 8.5, 2.5 Hz, 1H), 5.08 (s, 2H), 4.20 (s, 1H), 3.83 (s, 3H), 3.51 (s, 3H), 3.07–3.11 (m, 2H), 2.66–2.69 (m, 2H), 2.54–2.57 (m, 4H), 1.01–1.04 (m, 6H). ^13^C NMR (CDCl_3_): δ 151.3, 145.5, 138.5, 119.5, 104.3, 98.9, 96.9, 56.3, 55.9, 51.9, 46.9, 42.1, 11.9.

#### Step 2

A mixture of **14** (1.55 g, 9.77 mmol) and **17** (0.92 g, 3.25 mmol) in CH_3_CN (40 mL) was stirred at rt for 30 min before NaBH(OAc)_3_ (2.41 g, 11.4 mmol) was added. The mixture was stirred at rt for 16 h and then quenched by adding saturated NaHCO_3_. This was then extracted with EA (3 × 50 mL), washed with brine, dried over MgSO_4_, and concentrated to afford a residue that was purified by sg chromatography using hexane/EA/triethylamine (50/45/5) as an eluent to give *N*-[2-[(*tert*-butoxycarbonyl)ethylamino]ethyl]-*N*-[2-(diethylamino)ethyl]-3-methoxy-4-(methoxymethoxy)aniline (**18**) as an orange oil (1.1 g, 73%). ^1^H NMR (CDCl_3_): δ 6.99 (d, *J* = 8.5 Hz, 1H), 6.3 (d, *J* = 2.5 Hz, 1H), 6.22 (dd, *J* = 7.0, 2.5 Hz, 1H), 5.09 (s, 2H), 3.86 (s, 3H), 3.52 (s, 3H), 3.31–3.41 (m, 8H), 2.55–2.59 (m, 6H), 1.46 (s, 9H), 1.02–1.11 (m, 9H).

#### Step 3

NBS (0.46 g, 12.61 mmol) was added in portions with stirring over 10 min to a solution of **18** (0.79 g, 1.74 mmol) in 10% aqueous acetic acid (30 mL) at −15 °C. The mixture was stirred at −15 °C for 30 min and then for 12 h at rt. After 2 M NaOH (30 mL) solution was added, the mixture was extracted with DCM (3 × 30 mL). The combined organic layers were dried over MgSO_4_ and concentrated *in vacuo*. The residue was purified by sg chromatography using hexane/EA/triethylamine (50/45/5) as an eluent to afford *N*-[2-[(*tert*-butoxycarbonyl)ethylamino]ethyl]-*N*-[2-(diethylamino)ethyl]-2-bromo-5-methoxy-4-(methoxymethoxy)aniline (**19**) as a brown oil (0.67 g, 72%). ^1^H NMR (CDCl_3_): δ 7.32 (s, 1H), 6.79 (s, 1H), 5.16 (s, 2H), 3.85 (s, 3H), 3.51 (s, 3H), 3.12–3.25 (m, 9H), 2.48–2.53 (m, 6H), 1.45 (s, 9H), 1.02–1.05 (m, 3H), 0.97–0.99 (m, 6H).

#### Step 4

To a stirred solution of **19** (0.4 g, 0.75 mmol) in MeOH (5 mL) was added acetyl chloride (0.235 g, 3.0 mmol) at −60 °C. The mixture was stirred at −60 °C for 30 min and then for 12 h at rt. The mixture was treated with 2 M NaOH (10 mL) and then extracted with DCM (2 × 20 mL). The combined organic layers were dried over MgSO_4_, filtered, and concentrated *in vacuo* to give the free base of **4** (0.28 g, 95%). ^1^H NMR (CDCl_3_): δ 7.02 (s, 1H), 6.69 (s, 1H), 3.79 (s, 3H), 3.18–3.20 (m, 2H), 3.02–3.05 (m, 2H), 2.65–2.69 (m, 2H), 2.62–2.68 (m, 2H), 2.51–2.61 (m, 6H), 1.14–1.17 (m, 3H), 0.96–0.99 (m, 6H). ^13^C NMR (DMSO-*d*_6_): δ 147.2, 144.6, 140.4, 118.7, 114.2, 108.6, 55.9, 54.0, 53.8. 50.5, 46.9. 46.7, 43.5, 14.3, 11.2. To a solution of the free base of **4** (0.2 g, 0.514 mmol) in diethyl ether (20 L was added dropwise a solution of naphthalene-1,5-disulfonic acid (0.16 g, 0.566 mmol) in acetone (10 mL). The mixture was stirred at rt for 2 h, and the precipitate was filtered, washed with acetone (2 × 25 mL), and dried at 60 °C to afford **4** (0.34 g, 99%). Mp: 178–180 °C dec. ^1^H NMR (D_2_O): δ 8.84 (d, *J* = 8.5 Hz, 2H), 8.19 (d, *J* = 7.0 Hz, 2H), 7.71 (t, *J* = 7.5 Hz, 2H), 7.16 (s, 1H), 7.01 (s, 1H), 3.82 (s, 3H), 3.26 (t, *J* = 6.8 Hz, 2H), 3.19 (t, *J* = 5.8 Hz, 2H), 3.03–3.07 (m, 4H), 2.98–3.01 (m, 4H), 2.90 (t, *J* = 7.3 Hz, 2H), 1.20 (t, *J* = 7.3 Hz, 3H), 1.09 (t, *J* = 7.3 Hz, 6H). ^13^C NMR (D_2_O): δ 148.8, 145.1, 139.0, 137.8, 129.3, 129.0, 126.8, 126.2, 119.2, 115.0, 109.8, 56.2, 52.1, 49.8, 49.2, 48.0, 44.4, 43.2, 10.6, 8.2. Anal. Calcd for C_27_H_38_BrN_3_O_8_S_2_·H_2_O: C, 46.68; H, 5.80; N, 6.05. Found: C, 46.77; H, 5.36; N, 5.68.

### *N*,*N*-Bis[2-(ethylamino)ethyl]-2-bromo-4,5-dimethoxyaniline 1,5-naphthalenedisulfonate (**5**)

#### Step 1

A mixture of **14** (3.74 g, 20 mmol) and 3,4-dimethoxyaniline (20) (766 mg, 5 mmol) in CH_3_CN (30 mL) was stirred at rt for 30 min before NaBH(OAc)_3_ (5.30 g, 25 mmol) was added. The mixture was stirred at rt for 48 h and concentrated. The residue was diluted with saturated NaHCO_3_ (40 mL), stirred at rt for 30 min, and extracted with DCM (3 × 60 mL). The combined organic layers were washed with water (100 mL) and brine (50 mL), dried over MgSO_4_, filtered, and concentrated *in vacuo*. The crude product was purified by sg chromatography using hexanes/EA (7/3) as an eluent to afford *N*,*N*-bis[2-[(*tert*-butoxycarbonyl)ethylamino]ethyl]-3,4-dimethoxyaniline (**21**; 1.75 g, 71%).

#### Step 2

To a solution of **21** (1.75 g, 3.53 mmol) and Na_2_CO_3_ (1.75 g, 16.5 mmol) in DCM (50 mL) and water (50 mL) at rt was added NBS (900 mg, 5.06 mmol). The mixture was stirred at rt for 17 h. After separation of the organic layer, the aqueous layer was extracted with DCM (2 × 50 mL). The combined organic layers were washed with water (100 mL) and brine (50 mL), dried over MgSO_4_, filtered, and concentrated *in vacuo*. The crude product was purified by sg chromatography using hexanes/EA (7/3) as an eluent to afford *N*,*N*-bis[2-[(*tert*-butoxycarbonyl)ethylamino]ethyl]-2-bromo-4,5-dimethoxyaniline (**22**; 1.20 g, 59%). ^1^H NMR (CDCl_3_): δ 1.04 (br s, 6H), 1.43 (s, 18H), 3.01–3.39 (m, 12H), 3.84 (s, 3H), 3.88 (s, 3H), 6.85 (br s, 1H), 7.03 (s, 1H).

#### Step 3

To **22** (0.90 g, 1.57 mmol) was added a solution of methanesulfonic acid (2.88 g, 30 mmol) in ether (20 mL). The mixture was stirred at rt for 24 h and diluted with ether (50 mL). The top ether layer was decanted to leave an oily residue. The residue was treated with ether (50 mL), and the ether layer was decanted again. To the residue was added a solution of 1,5-naphthalenedisulfonic acid (435 mg, 1.57 mmol) in THF (30 mL). After the mixture was stirred at rt for 1 h, the resulting precipitate was filtered and dried to afford **5** (971 mg, 93%) as a white solid. Mp: 144–146 °C. ^1^H NMR (DMSO-*d*_6_): δ 1.12 (t, *J* = 7 Hz, 6H), 2.81–3.01 (m, 8H), 3.20 (t, *J* = 7 Hz, 4H), 3.75 (s, 3H), 3.76 (s, 3H), 7.04 (s, 1H), 7.16 (s, 1H), 7.46 (t, *J* = 8 Hz, 2H), 7.98 (d, *J* = 7 Hz, 2H), 8.22 (s, 4H), 8.89 (d, *J* = 9 Hz, 2H). ^13^C NMR (DMSO-*d*_6_): δ 11.18, 42.41, 43.92, 50.21, 56.20, 110.73, 112.40, 115.86, 124.28, 124.36, 129.27, 129.66, 139.42, 143.69, 147.61, 149.08. Anal. Calcd for C_26_H_36_BrN_3_O_8_S_2_: C, 47.13; H, 5.48; N, 6.34. Found: C, 47.06; H, 5.60; N, 6.12.

### *N*,*N*-Bis[2-(diethylamino)ethyl]-2-bromo-5-hydroxy-4-methoxyaniline trihydrobromide (**6**)

#### Step 1

To a stirred mixture of 4-methoxy-3-(methoxymethoxy)aniline (**10**;^26^ 2.0 g, 10.91 mmol) and K_2_CO_3_ (15.09 g, 109.16 mmol) in anhydrous DMF (30 mL) at rt was added in portions 2-diethylaminoethyl chloride hydrochloride (15.03 g, 87.33 mmol). The reaction mixture was stirred for 40 h at 145 °C. The reaction mixture was cooled to rt, poured into water (100 mL), and extracted with EA (3 × 50 mL). The organic layer was washed with water (3 × 50 mL), dried over MgSO_4_, filtered, and concentrated *in vacuo*. The residue was purified by sg chromatography using hexane/EA/triethylamine (50/45/5) as an eluent to give *N*,*N*-bis[2-(diethylamino)ethyl]-4-methoxy-3-(methoxymethoxy)aniline (**11**; 3.16 g, 76%). ^1^H NMR (CDCl_3_): δ 6.80 (d, *J* = 9.26 Hz, 1H), 6.64 (d, *J* = 2.92, 1H), 6.29 (dd, *J* = 9.26, 2.92 Hz, 1H), 5.19 (s, 2H), 3.79 (s, 3H), 3.49 (s, 3H), 3.34 (t, *J* = 7.8 Hz 4H), 2.54–2.59 (m, 12H), 1.04 (t, *J* = 7.0, 12H). ^13^C NMR (CDCl_3_): δ 158.1, 143.8, 140.8, 114.2, 105.4, 102.3, 95.8, 56.8, 56.0, 50.5, 50.2, 47.6, 11.8

#### Step 2

Bromine (0.48 g, 3.01 mmol) in acetic acid (2 mL) was added dropwise with stirring over 30 min to a solution of **11** (1.0 g, 2.6 mmol) in 10% aqueous acetic acid (40 mL) at 0 °C. The mixture was stirred for 1 h at 0 °C and then for 12 h at rt. To the reaction mixture was added 2 M NaOH (20 mL) followed by extraction with DCM (2 × 20 mL). The combined organic layers were dried over MgSO_4_, filtered, and concentrated *in vacuo*. The residue was purified by sg chromatography using hexane/EA/triethylamine (50/45/5) as an eluent to afford *N*,*N*-bis[2-(diethylamino)ethyl]-2-bromo-4-methoxy-5-(methoxymethoxy)aniline (**12**; 0.60 g, 50%). ^1^H NMR (CDCl_3_): δ 7.08 (s, 1H), 7.06 (s, 1H), 5.17 (s, 2H), 3.85 (s, 3H), 3.48 (s, 3H), 3.05–3.07 (m, 4H), 2.45–2.50 (m, 12H), 0.98 (t, *J* = 7.3, 12H).

#### Step 3

To a stirred solution of **12** (1.1 g, 2.38 mmol) in MeOH was added acetyl bromide (1.17 g, 9.55 mmol) at −60 °C. The mixture was stirred at −60 °C for 30 min and then for 12 h at rt. Solvent removal *in vacuo* afforded **6** (0.98 g, 99%). Mp: 85–87 °C. ^1^H NMR (DMSO-*d*_6_): δ 9.38 (s, 2H), 7.14 (s, 1H), 6.89 (s, 1H), 3.76 (s, 3H), 3.35–3.38 (m, 4H), 3.11–3.17 (m, 12H), 1.16 (t, *J* = 7.3, 12H). ^13^C NMR (DMSO-*d*_6_): δ 147.2, 146.5, 139.8, 116.9, 112.8, 109.8, 56.7, 48.5, 47.3, 9.1. Anal. Calcd for C_19_H_37_Br_4_N_3_O_2_: C, 34.62; H, 5.66; N, 6.38. Found: C, 34.39; H, 5.38; N, 6.20.

### *In Vitro* Metabolic Stability

Following the protocols described by Charman et al.,^28^**1** was incubated at 37 °C with human, rat, and rhesus monkey liver microsomes. For the determination of intrinsic clearance, the substrate concentration was 1 μM and the protein concentration was 0.4 mg/mL; for qualitative metabolite identification studies, the substrate and protein concentrations were 10 μM and 1 mg/mL, respectively. The reaction was initiated by the addition of an NADPH-regenerating buffer system, and the samples were incubated for up to 60 (for Clint determination) or 180 min (for additional metabolite identification). Samples without the test compound and without NADPH were also incubated and used as controls. The reaction was quenched by protein precipitation with the addition of an equal volume of ice-cold acetonitrile solution (containing diazepam as an internal standard), followed by vortexing and centrifugation for 3 min at 10000 rpm. The supernatant was removed and analyzed by LC/MS. LC/MS analysis was conducted using a Waters Micromass Xevo G2 QTOF MS coupled to a Waters Acquity UPLC. For metabolite identification, a Supelco Ascentis Express Amide column (50 × 2.1 mm, 2.7 μm) was used with an acetonitrile–water gradient (containing 0.05% formic acid), a flow rate of 0.4 mL/min, a gradient cycle time of 6 min, and an injection volume of 5 μL. MS analysis was conducted using positive mode electrospray ionization under MSE acquisition mode, which allows simultaneous acquisition of MS spectra at low and high collision energies. The identity of putative metabolites was confirmed by the accurate mass and a comparison of the retention times and MS/MS fragmentation patterns with authentic metabolites where available. For metabolite quantitation, the method described below for the analysis of plasma samples was used.

### Pharmacokinetics

Animal studies were conducted using established procedures in accordance with the Australian Code of Practice for the Care and Use of Animals for Scientific Purposes, and the study protocols were reviewed and approved by the Monash Institute of Pharmaceutical Sciences Animal Ethics Committee. The study was conducted in overnight-fasted male Sprague–Dawley rats weighing 266–292 g. Rats had access to water *ad libitum* throughout the pre- and postdose sampling period, and access to food was reinstated 4 h postdose. Compound **1** was administered intravenously as a 10 min constant rate infusion via an indwelling jugular vein cannula (1 mL per rat, *n* = 5 rats) and orally by gavage (10 mL/kg per rat, *n* = 2–3 rats), and samples of arterial blood and total urine were collected up to 24 h postdose. Arterial blood was collected directly into borosilicate vials (at 4 °C) containing heparin. Blood samples were centrifuged, and the supernatant plasma was removed and stored frozen (−80 °C) until analysis by LC-MS. Plasma samples and calibration standards (prepared in blank rat plasma) were processed by protein precipitation with a 2-fold volume of acetonitrile. LC-MS analysis was conducted using a Waters Micromass Quattro Premier triple-quadrupole mass spectrometer coupled to a Waters Acquity UPLC with a Phenomenex C18 Kinetex column (50 × 2.1 mm, 2.7 μm). Elution was achieved using a methanol–water gradient (containing 8.2 mM formic acid and 1.25 mM ammonium formate) with a flow rate of 0.6 mL/min, a gradient cycle time of 6 min, and an injection volume of 2 μL. MS detection was conducted using positive electrospray ionization with multiple reaction monitoring. Transitions (*m*/*z*), cone voltages (V), and CID voltages (V) were as follows: **1**, 430.26 > 99.93, 40, 20; **2**, 416.27 > 99.80, 30, 20; **3**, 402.22 > 99.93, 35, 20; **4**, 388.23 > 99.86, 35, 20; **5**: 374.25 > 72.01, 35, 25; diazepam (285.25 > 1554.09, 40, 25) (used as an internal standard). The quantitation range in plasma was typically 1–10000 ng/mL, and the accuracy and precision were within ±10% and <10%, respectively, for each analyte. Urine samples were analyzed using either a 20- or 200-fold dilution with 50% acetonitrile and assayed against calibration standards prepared in the same solvent.

### *P. vivax* Liver-Stage *In Vitro* Assay

Compounds were prepared as 20 mM stock solutions in DMSO and tested in an eight-point semilog (1/3) dilution series from 20 μM in 384-well assay plates in both prophylactic and radical cure mode against *P. vivax* liver-stage parasites as previously described.^16^ Following ethical approval from the Institutional Ethics Committee of the Thai Ministry of Public Health, the Ethical Review Committee of Faculty of Tropical Medicine, Mahidol University (TMEC 11-008 and 14-016), the Oxford Tropical Medicine Ethical Committee, Oxford University, England (OxTREC 17-11 and 40–14), and the Cambodian National Ethics Committee for Health Research (101NECHR), isolates of *P. vivax* infected blood were collected from patent volunteers in Tak province, Thailandm and Mondulkiri province, Cambodia. Samples were collected by venipuncture into heparin tubes prior to replacement of patient serum with nonimmune AB human serum and feeding to laboratory-reared *Anopheles dirus* cracens or *An. dirus* A mosquitoes via water-jacket artificial membrane feeders or the Hemotek insect feeding system (Hemotek, Blackburn, UK) set to maintain the bloodmeal temperature at 37 °C. Salivary gland sporozoites were dissected from mosquitoes at day 14–18 postbloodmeal and collected into RPMI lacking sodium bicarbonate (Gibco, Thermo Fisher Scientific). Cryopreserved human hepatocytes (BioIVT, Baltimore, Maryland, USA) were thawed into InVitroGro CP Medium (BioIVT), quantified for viability by trypan blue dye exclusion, diluted to 800–1000 live cells per microliter, and 18000 live cells were plated into each well of a collagen-coated 384-well plate (Greiner, Monroe, NC, USA) by a 16-channel pipet (Finnpipette, Thermo Fisher Scientific, Waltham, MA). After 2 days in culture, hepatocyte-containing wells were infected with 5000–20000 freshly dissected salivary gland sporozoites diluted into plate media. Compounds were plated into 384-well plates (Greiner) at 1000×x final concentration in DMSO. A custom-manufactured pin tool (V&P Scientific, San Diego, CA, USA) was used to transfer 40 nL of the compound in DMSO into the assay plate with 40 μL of media and infected hepatocytes, thereby delivering a 1000-fold dilution of each drug at each dose. Prophylactic plates were treated on days 1–4 post sporozoite infection, and radical cure plates were treated on days 5–7 post sporozoite infection. The medium was changed every other day outside of the treatment window and prior to treatment on each day of the treatment window. Prophylactic plates were fixed on day 6 postinfection and radical cure plates were fixed on day 8 postinfection addition with 4% paraformaldehyde in PBS. Fixed plates were stained with 40 ng/mL mouse antirecombinant *P. vivax* Upregulated in Infectious Sporozoites 4 (rUIS4)^29^ in a permeabilizing and blocking dilution buffer (0.03% Triton X-100 and 1% BSA in PBS) overnight at 4 °C. Plates were then washed three times with PBS and stained with 2 μg/mL of goat antimouse Alexafluor 488 conjugated secondary antibody (Thermo Fisher Scientific) in dilution buffer overnight at 4 °C. Plates were then washed three times with PBS, stained with 10 μg/mL of Hoechst in PBS for 1 h, washed again, and sealed for imaging. Plates were imaged on an ImageXpress Micro high content imaging system, and images were analyzed with MetaXpress software (Molecular Devices, Sunnyvale, CA, USA). Data including form-specific parasite counts and parasite size per well were normalized to DMSO negative control and ionophore positive control, and IC_50_ values were calculated in CDD Vault (Burlingame, CA, USA). The full protocol for *P. vivax* assays is available; the radical cure format used was the 8 day version 2 assay.^30^

### *P. cynomolgi* Liver-Stage *In Vitro* Assay

Briefly, rhesus monkey hepatocytes were isolated from liver lobes as described by Guguen-Guillouzo et al.^31^ Sporozoite infections were performed within 3 days after hepatocyte isolation. Sporozoite inoculation of primary rhesus hepatocytes was performed according to the method of Dembele et al.^32^ Hepatocytes were washed with William’s B medium (William’s E + Glutamax plus 10% human serum (AB+), 1% insulin/transferrin/selenium, 1% sodium pyruvate, 1% MEM-NEAA, 2% Pen/strep, 0.05 μM hydrocortisone, 50 μM 2-mercaptoethanol) before adding 50000 sporozoites/well. Compound treatments were started at the first medium refreshment after sporozoite inoculation (prophylactic assay mode). Compounds were diluted in William’s B medium to 10, 1, and 0.1 μM. Controls were included in every assay plate. Assay plates were fixed at day 6 postinfection with 4% PFA and stained with antibodies for high content screening. Antibodies (1:10000 Anti Hsp70.1 polyclonal (rabbit) and 1:1000 Alexa 588-labeled Goat-anti Rabbit-IG) were diluted in in 0.03% Triton X-100, 1% (w/v) BSA in 1 × PBS were used for visualization of intracellular parasites.^16^ An Operetta-based analysis (PerkinElmer) was performed as described previously,^20^ differentially counting small and large liver-stage parasites.

## Abbreviations

ADME: absorption–distribution–metabolism–excretion
DCM: dichloromethane
DIPEA: diisopropylethylamine
DMF: dimethylformamide
EA: ethyl acetate
MOM: methoxymethyl
NBS: *N*-bromosuccinimide
